# Stress and anxiety during pregnancy and length of gestation: a federated study using data from five Canadian and European birth cohorts

**DOI:** 10.1007/s10654-024-01126-4

**Published:** 2024-05-28

**Authors:** Julie Bergeron, Demetris Avraam, Lucinda Calas, William Fraser, Jennifer R. Harris, Barbara Heude, Piush Mandhane, Theo J. Moraes, Gina Muckle, Johanna Nader, Jean R. Séguin, Elinor Simons, Padmaja Subbarao, Morris A. Swertz, Suzanne Tough, Stuart E. Turvey, Isabel Fortier, Naja Hulvej Rod, Anne-Marie Nybo Andersen

**Affiliations:** 1 Department of Public Health, University of Copenhagen, Copenhagen, Denmark; 2 Research Institute of the McGill University Health Center, Montreal, Canada; 3 Population Health Sciences Institute, Newcastle University, Newcastle Upon Tyne, UK; 4 Centre for Research in Epidemiology and Statistics, INSERM, Paris, France; 5 Department of Obstetrics and Gynecology, Université de Sherbrooke, Sherbrook, Canada; 6 Centre for Fertility and Health, The Norwegian Institute of Public Health, Oslo, Norway; 7 Department of Pediatrics, University of Alberta, Edmonton, Canada; 8 Department of Paediatrics, Hospital for Sick Children and University of Toronto, Toronto, Canada; 9 School of Psychology, Université Laval, Quebec, Canada; 10 Department of Psychiatry and Addictology, Université de Montréal and CHU Ste-Justine Research Center, Montreal, Canada; 11 Department of Pediatrics and Child Health, University of Manitoba, Winnipeg, Canada; 12 Department of Genetics, University Medical Center Groningen, University of Groningen, Groningen, the Netherlands; 13 Department of Paediatrics, University of Calgary, Calgary, Canada; 14 Department of Pediatrics, BC Children’s Hospital, University of British Columbia, Vancouver, Canada

**Keywords:** Gestational age at birth, Perceived stress, Anxiety, Multi cohort study, Data harmonization

## Abstract

While its etiology is not fully elucidated, preterm birth represents a major public health concern as it is the leading cause of child mortality and morbidity. Stress is one of the most common perinatal conditions and may increase the risk of preterm birth. In this paper we aimed to investigate the association of maternal perceived stress and anxiety with length of gestation. We used harmonized data from five birth cohorts from Canada, France, and Norway. A total of 5297 pregnancies of singletons were included in the analysis of perceived stress and gestational duration, and 55,775 pregnancies for anxiety. Federated analyses were performed through the DataSHIELD platform using Cox regression models within intervals of gestational age. The models were fit for each cohort separately, and the cohort-specific results were combined using random effects study-level meta-analysis. Moderate and high levels of perceived stress during pregnancy were associated with a shorter length of gestation in the very/moderately preterm interval [moderate: hazard ratio (HR) 1.92 (95%CI 0.83, 4.48); high: 2.04 (95%CI 0.77, 5.37)], albeit not statistically significant. No association was found for the other intervals. Anxiety was associated with gestational duration in the very/moderately preterm interval [1.66 (95%CI 1.32, 2.08)], and in the early term interval [1.15 (95%CI 1.08, 1.23)]. Our findings suggest that perceived stress and anxiety are associated with an increased risk of earlier birth, but only in the earliest gestational ages. We also found an association in the early term period for anxiety, but the result was only driven by the largest cohort, which collected information the latest in pregnancy. This raised a potential issue of reverse causality as anxiety later in pregnancy could be due to concerns about early signs of a possible preterm birth.

**Supplementary Information:**

The online version contains supplementary material available at 10.1007/s10654-024-01126-4.

## Introduction

Approximately 11% of the children worldwide are born preterm [1]. Preterm birth is the leading cause of death of children under the age of five years globally [2, 3], and children born preterm are at higher risk of serious health and developmental problems, including cerebral palsy, cognitive impairment, respiratory diseases and vision and hearing impairment [1, 4–7]. While the risk of morbidity and mortality is the highest in extremely preterm born children (22–27 weeks), late preterm (34–36 weeks) and even early term (37–38 weeks) born children also have higher risks of adverse outcomes compared to full term born children [5, 8–10]. The risk factors for preterm birth are many, including delayed childbearing, obesity, ascending infections, severe pregnancy complications such as preeclampsia and abruption of the placenta, and physically demanding work including lifting of heavy burdens [1, 3, 11, 12], but much remains to be understood regarding preterm etiology. A strong social and, particularly in the United States, racial inequalities gradient in preterm birth [13–17] has consistently been found, which has led to the suggestion that maternal stress exposure might be an etiologic factor for preterm birth.

Pregnancy is a period of physiological and psychological change, which may induce or reinforce stress and anxiety [18, 19]. Pregnant women also seem more susceptible to the impact of stress [20, 21]. Prenatal psychosocial stress encompasses a variety of features, including daily life stressors, major life events and anxiety [15, 22, 23]. A potential mechanism through which stress and anxiety may impact length of gestation is by activating inflammatory processes or immune responses [24–26]. Indeed, psychosocial stress has been shown to increase levels of corticotropin-releasing hormone and oxytocin, as well as proinflammatory markers (e.g. cytokines, prostaglandins), all of which have been associated with the initiation of parturition by stimulating uterine contractions [27–30]. Findings from some past studies indicate that women experiencing stress and anxiety during pregnancy are at higher risk of adverse birth outcomes [31–35], while other studies have not found this association [32, 36]. Small sample sizes, differences in stress measurements used, timing of assessments of stress in pregnancy, and different contexts may contribute to the inconsistencies in the results.

The EUCAN connect [37] initiative brings together researchers and existing birth cohort consortia in Europe and Canada to promote reuse of already collected data and to develop methods and software enabling data harmonization and federated analyses. By leveraging the co-analysis of Canadian and European birth cohorts, this initiative provides a unique opportunity to investigate research questions about rare outcomes, such as being born very preterm, because co-analyzing data from multiple cohorts provides larger sample sizes and increase statistical power. By facilitating co-analysis across international cohorts, the project also allows comparison of results between different contexts and populations. In this study, we aim to assess the associations between maternal perceived stress or anxiety and gestational duration by making use of the data collected in five mother and child cohorts across Canada, France, and Norway, which have been harmonized and made suitable for federated analyses in the EUCAN Connect initiative [37] and related projects (LifeCycle project [38, 39] and Research Advancement through Cohort Cataloguing and Harmonization (ReACH) initiative [40]).

## Methods

### Participating cohorts and selection of participants

Pregnancy cohorts from Canada were identified through the Research Advancement through Cohort Cataloguing and Harmonization (ReACH) initiative [40] and those from Europe were identified through the LifeCycle project catalogue [38]. Eligible cohort studies had to include information about maternal stress or anxiety during pregnancy, collected using standardized questionnaires. They also needed to have recruited the mothers during pregnancy, followed them until delivery and have information about gestational age at birth. Five cohorts fulfilling these criteria were selected, three from Canada (3D Study—Design, Develop, Discover (3D) [41], All Our Families (AOF) [42] and CHILD Cohort Study (CHILD) [43]), one from France (EDEN—Study on the pre and early postnatal determinants of child health and development (EDEN) [44]) and one from Norway (Norwegian Mother, Father and Child Cohort Study (MoBa) [45]) (Table 1). We approached the principal investigators of each cohort to invite them to participate in this study and all five accepted.

**Table 1:** Characteristics of the participating cohorts

Cohort	Country	Start year	Recruitment	Number of mothers	Measure of stress and anxiety
3D study—Design, Develop, Discover (3D)	Canada	2010	8–14 weeks pregnant	2,366	PSS
All Our Families (AOF)	Canada	2008	< 25 weeks pregnant	3,387	PSS, STAI
CHILD Cohort Study (CHILD)	Canada	2008	Second or third trimester	3,412	PSS
EDEN—Study on the pre and early postnatal determinants of child health and development (EDEN)	France	2003	24–28 weeks pregnant	2,002	STAI
Norwegian Mother, Father and Child Cohort Study (MoBa)	Norway	1999	13–17 weeks of pregnancy	89,283	SCL

*PSS* Perceived Stress Scale, *STAI* State-Trait Anxiety Inventory, *SCL* Symptom Checklist

In this study, pregnancies from the selected cohorts were included if the women were expecting a singleton baby (n = 97,320). Only the first pregnancy recorded during the follow-up of the cohorts was selected (n = 81,265). Pregnancies were eligible if the mother completed either the stress or anxiety questionnaires at least once between week 13 and 32 of pregnancy. In total, 6646 pregnancies from three cohorts (3D, AOF and CHILD) were included in the analyses regarding stress and gestational duration, and 61,173 pregnancies, also from three cohorts (AOF, EDEN and MoBa), were included in the analyses regarding anxiety and length of gestation (Figure S1).

### Gestational age at birth

Information on gestational age at birth was retrieved in all cohorts from the mother’s medical chart and was based on a combination of results from ultrasound scans and estimated date of delivery based on date of last menstrual period. Gestational age at birth was harmonized as a continuous variable in days. In AOF, the gestational age was only recorded in weeks. To obtain the gestational age in days, we performed imputation using the distribution of births within each week from the Danish National Birth Cohort [46], chosen due to its large size, and randomly assigned women in AOF a number of days within the gestational week following that distribution.

### Stress and anxiety

Stress during pregnancy was measured using the Perceived Stress Scale (PSS) short form [47] in three of the participating cohorts (3D, AOF, CHILD). The PSS short form comprises 4 items, as listed in Table S1, with a total score ranging from 0 to 16. We wanted to compare groups of women with different levels of stress, but since the PSS has no standardized cut-offs, the pregnant women were divided into three groups according to tertiles (T1 = Low stress, T2 = Moderate stress, T3 = High stress). Anxiety during pregnancy was collected in three of the cohorts, using the State Trait Anxiety Inventory (STAI) [48] in AOF and EDEN and with a short form of the Symptoms Checklist (SCL-8) [49] in MoBa. The STAI has a standardized cut-off at a score of 40, and this was used to dichotomize the women into two groups, those with a score above the cut-off and those with a score below. In both cohorts using the STAI data, around 16% of the women had a score of 40 and above. Since the SCL-8 does not have a specified cut-off, we used a cut-off that dichotomized the scores of the women into groups with the most similar distribution as had been found in cohorts using the STAI, resulting in 11% of women being identified as having scores above the cut-off. If a cohort had more than one time of measurement of stress or anxiety during the interval of 13 to 32 weeks of pregnancy, the earliest measure was used.

### Covariates

Potential confounders were selected using the method of directed acyclic graph (Figure S2). Some of the potential confounders identified from the literature were however omitted from the analyses due to the information not being collected by all cohorts (fertility treatment, infections during pregnancy and the child’s congenital anomalies). In the end, the potential confounders that could be generated across all five cohorts were the mother’s age at delivery, highest level of education, living with a partner, parity and pregnancy complications. Age of the mothers at delivery was defined in years. Highest attained level of education was categorized according to the International Standard Classification of Education (ISCED) [50] as low (ISCED 0–2), medium (ISCED 3–4) or high (ISCED 5–8). Living with a partner during pregnancy was dichotomized as “yes” or “no”. Parity was dichotomized as primiparous or parous. Finally, maternal pregnancy complications were defined as having any of the following self-reported conditions during pregnancy: hypertension, preeclampsia, or diabetes.

### Harmonization process

To allow for co-analysis of data across the five cohorts, the data had to be harmonized under a common format. For the European cohorts, a core set of variables had already been harmonized as part of the LifeCycle project [39]. From the extensive list of variables generated in the LifeCycle project, the outcome, exposures and potential confounders described above were selected. Additional harmonization was conducted by the cohorts’ team to generate the stress or anxiety scores and a variable about the timing of data collection during pregnancy. The LifeCycle variable definitions were then used to guide the harmonization of the Canadian cohorts’ data. In Canada, the harmonization was performed centrally by the Maelstrom Research team following the Maelstrom guidelines for retrospective harmonization [51]. In Figure S3, the algorithmic transformation to generate the highest level of education from the information collected by the five cohorts is provided as an example illustrating the harmonization process.

### Statistical analyses

The analyses were conducted using DataSHIELD [52], which is a platform developed to allow privacy-preserving remote federated analyses, without the need of moving the data outside of their original hosting servers. Through DataSHIELD, the analyst cannot extract or see any individual-level data, but only receives non-disclosive summary statistics from commands requested for specific statistical analyses. Using the dsSurvival package (version 1.0.0) [53] in DataSHIELD, two sets of Cox regressions were performed, one on the association between levels of perceived stress and gestational duration and one on the association between anxiety and gestational age at birth. The underlying time scale was the gestational age in days. The event analyzed was giving birth, regardless of the child being liveborn or stillborn. Women entered the study on the day they completed the stress or anxiety questionnaire, so delayed entry was applied in the models. The follow-up ended at the time of birth. All analyses were performed only for complete cases for the set of variables in each model, due to the absence of imputation methods in DataSHIELD. The proportional hazard assumption on the full range of gestational ages was tested using the Schoenfeld residuals and, since it was not met for both exposures, separate analyses were performed according to intervals of gestational ages. Thus, hazard ratios for the rate of giving birth very or moderately preterm (24–33 completed weeks), late preterm (34–36 completed weeks), early term (37–38 completed weeks) and full term (39–42 completed weeks) were obtained for the different levels of stress or anxiety. Four sets of analyses were thus performed for both perceived stress and anxiety. Through DataSHIELD, the models were fitted separately for each cohort, and the cohort-specific regression coefficients and standard errors were then combined using random effects (RE) study-level meta-analysis with the restricted maximum likelihood estimator method [54]. Heterogeneity between cohorts was assessed using the I^2^ and the chi-squared Q statistics.

### Sensitivity analysis

Due to the high proportion of missing values for some of the covariates, we performed a set of sensitivity analyses, removing the covariates “cohabitation status” and “maternal pregnancy complications” one at a time as these particularly had many missing values in some cohorts, up to 17% and 41% respectively.

## Results

**Fig. 1:**
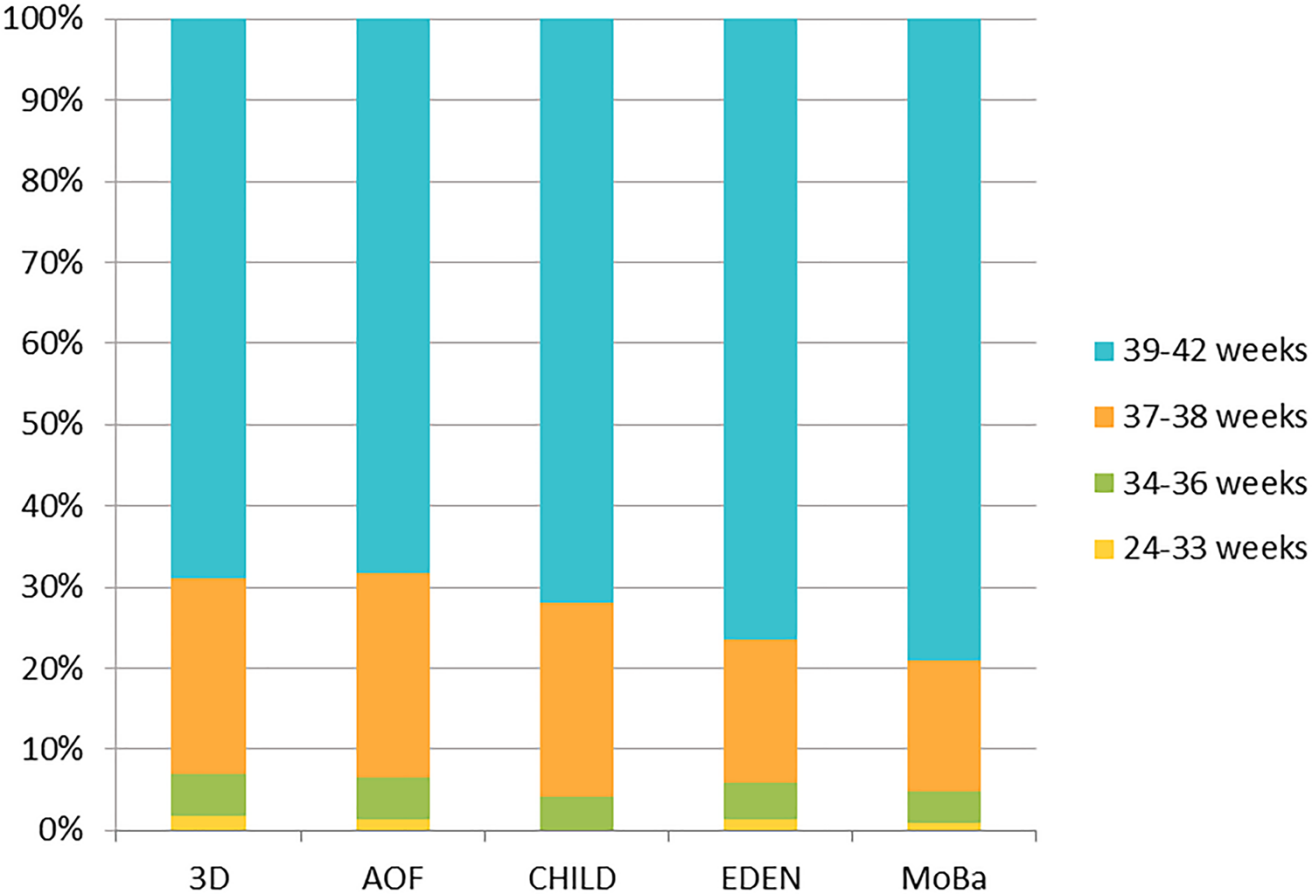
Distribution of births per interval of gestational age in participating cohorts

The prevalence of births per interval of gestational age is shown in Fig. 1 for the participating cohorts. Table 2 presents the characteristics of participants from each cohort. The characteristics of mothers were mostly similar across the different cohorts. However, an important difference relates to the timing of the stress and anxiety measurement, ranging from an average of 18 weeks of pregnancy for AOF to 30 weeks for MoBa.

**Table 2:** Participant characteristics by cohorts

Characteristics	Perceived stress				Anxiety			
	All (n = 6,646)	3D (n = 1,787)	AOF (n = 2,610)	CHILD (n = 2,249)	All (n = 61,173)	AOF (n = 2,531)	EDEN (n = 1,653)	MoBa (n = 56,989)
*Age* [mean (SD)]	31.6 (4.5)	31.5 (4.5)	31.2 (4.4)	32.0 (4.6)	29.9 (4.6)	31.3 (4.4)	29.5 (4.9)	29.9 (4.5)
*Education levels*								
Low (%)	2.8	1.3	3.7	2.9	2.5	3.7	6.3	2.3
Medium (%)	23.6	32.5	21.8	18.5	31.5	21.7	37.7	31.7
High (%)	72.7	64.2	74.3	77.8	60.4	74.5	55.1	59.9
Missing (%)	0.9	2.0	0.2	0.8	5.6	0.2	0.9	6.0
*Living with a partner*								
No (%)	2.4	2.5	0.9	4.1	3.4	0.9	5.4	3.5
Yes (%)	86.9	80.9	85.6	93.2	94.7	86.0	94.4	95.1
Missing (%)	10.7	16.6	13.5	2.7	1.9	13.1	0.1	1.4
*Parity*								
0 (%)	50.7	56.5	45.6	52.1	55.9	46.0	45.7	56.6
1 + (%)	44.2	43.5	41.2	47.8	42.9	41.7	54.1	42.6
Missing (%)	5.1	0.0	12.9	0.1	1.2	12.3	0.2	0.8
*Pregnancy complications*								
No (%)	86.1	79.4	87.7	89.7	62.3	87.6	48.2	93.7
Yes (%)	13.4	19.5	12.3	9.8	6.0	12.4	10.4	5.5
Missing (%)	0.5	1.1	0.0	0.4	1.8	0.0	41.3	0.7
*Perceived stress*								
Low (%)	33.1	27.1	39.8	30.1				
Moderate (%)	35.3	40.3	27.9	40.1				
High (%)	31.6	32.6	32.3	29.9				
*Anxiety*								
No (%)					88.7	83.6	83.7	89.0
Yes (%)					11.3	16.4	16.3	11.0
*Gestational age at stress/anxiety measurement* (days) [mean (SD)]	146.1 (33.4)	137.2 (25.4)	128.3 (23.3)	173.7 (31.0)	206.4 (18.4)	128.2 (23.1)	183.0 (10.9)	210.6 (5.5)

### Perceived stress and length of gestation

**Fig. 2:**
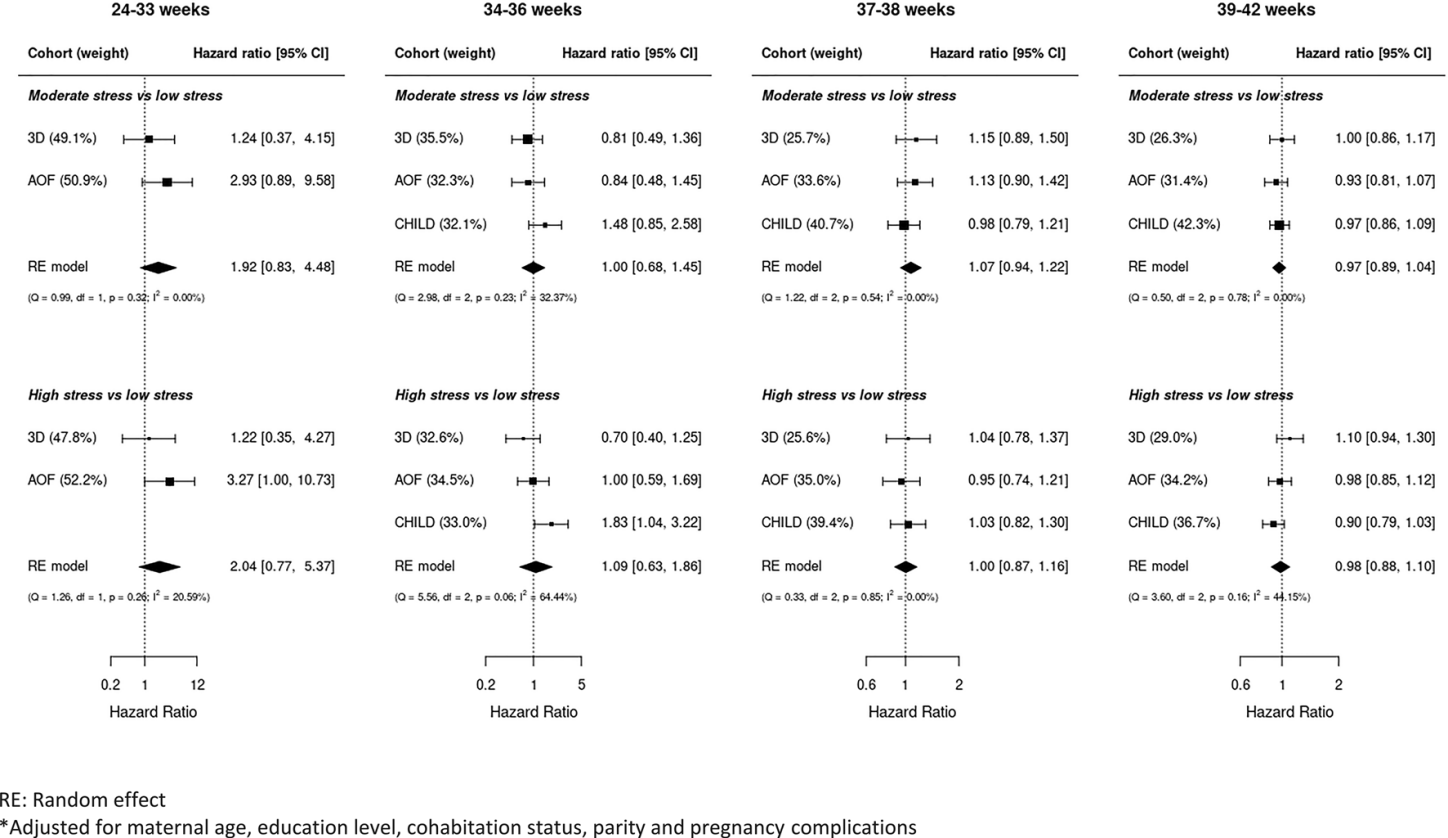
Forest plot of the association between perceived stress and the rate of giving birth in intervals of gestational age*

The complete case analysis for the association between perceived stress and gestational duration included 5,297 women (Figure S1). Figure 2 shows the adjusted hazard ratios of the association between levels of perceived stress and gestational age at birth in days in different intervals of gestational age. While only few (n = 41) births occurred before 34 weeks of pregnancy, the estimates indicated an association between moderate (HR = 1.92; 95%CI 0.83; 4.48) and high (HR = 2.04; 95% CI 0.77; 5.37) levels of stress with length of gestation, albeit not statistically significant. At 34 weeks and beyond, the rate of birth was not associated with higher stress levels. For both moderate and high levels of stress, the heterogeneity between cohorts was the highest in the late preterm period (moderate: I^2^ = 32.4%; high: I^2^ = 64.4%).

### Anxiety and length of gestation

**Fig. 3:**
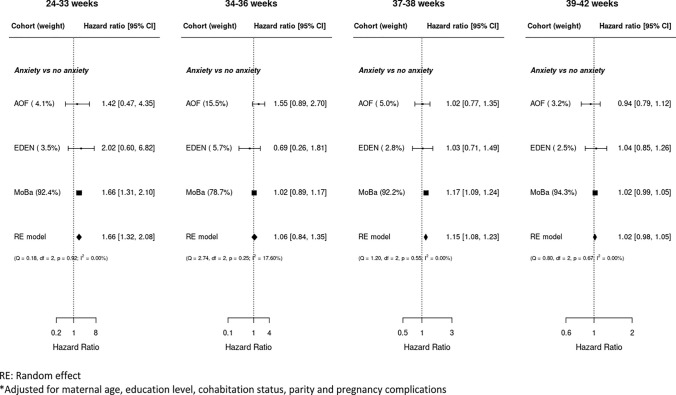
Forest plot of the association between anxiety and the rate of giving birth in intervals of gestational age*

There was information on the full set of variables for 55,775 women in the analyses of maternal anxiety and gestational age a birth (Figure S1). The results are presented in Fig. 3. Reporting anxiety was associated with lower gestational age at birth in the very to moderately preterm interval compared to women without clinical anxiety (HR = 1.66; 95% CI 1.32; 2.08). The meta-analyzed results also showed an association between anxiety and rate of birth in the early term interval, mainly driven by the results from the large MoBa birth cohort, while the other two cohorts showed no such association. The heterogeneity across cohorts was low across all gestational age intervals (I^2^ varying from 0.0% to 17.6%).

### Sensitivity analysis

Due to a high degree of missing information on cohabitation status and the mother’s pregnancy complications, we conducted a sensitivity analysis without adjustment of for these variables. The larger sample size and the lack of adjustment for these variables led to change in the estimates obtained for either the association between perceived stress or anxiety with gestational age at birth (Figures S4-7). Specifically, cohabitation status impacted the estimates for perceived stress and the mother’s pregnancy complications impacted the estimates for anxiety, especially in the earliest interval of gestational ages. To assess if these changes were the result of confounding or selection bias, we also performed the sensitivity analyses in the restricted sample (Figures S8-11). As the estimates obtained in the restricted sample were essentially the same as the ones in the non-restricted sample, the changes are most likely driven by confounding. Thus, the cohabitation status and pregnancy complications were kept as covariates in the main analyses.

## Discussion

### Principal findings

In this study, we observe an association between moderate and high levels of perceived stress and a higher rate of giving birth in the very to moderate preterm interval (24–33 weeks) when compared to women with a low level of stress. This association was, however, statistically uncertain due to few births within this interval. Having anxiety was also found to be associated with the length of gestation in the very to moderate preterm interval. A slightly higher rate of giving birth in the early term interval (37–38 weeks) was also observed for women with anxiety.

### Strengths and limitations

This study included harmonized data from five cohorts in Canada, France and Norway, which resulted in a large sample size and thus an opportunity to study rare outcomes such as births before week 32. It also allowed for exploration of the consistency of results in different settings and across populations. Investigating both a measure of perceived stress and one of anxiety during pregnancy is another important strength of this study as their association with the length of gestation has previously been found to differ [32, 33]. The nature of the exposure, i.e. stress and anxiety, is a subjective measure, however the use of validated questionnaires completed prospectively to assess maternal stress and anxiety ensured temporality of measurement between exposures and outcome with valid measurements. Finally, the variation of the timing of assessment for the stress and anxiety measures between cohorts is a strength of this study as some previous research showed a different impact of stress and anxiety on length of gestation according to when in pregnancy it is experienced [55–57]. The cohort specific results allowed the observation of possible variations in the association between stress or anxiety and the rate of giving birth depending on the periods of pregnancy when it was measured.

There are also some limitations to this study, the first one being inherent to the harmonization process. To be able to include all cohorts in the analysis, some confounders not collected by all cohorts had to be excluded (fertility treatment, infections during pregnancy and the child’s congenital anomalies) or the level of details of variable definitions adjusted to be more inclusive (e.g., the pregnancy complications included had to be limited to diabetes, hypertension and preeclampsia). While this trade off between quality of the information vs quantity of participants included is always at the forefront of projects co-analyzing data from multiple cohorts, it is important to note that these unmeasured confounders possibly biased our results. Due to the complexity of the harmonization work and the current limitations in analyses available in DataSHIELD, it was not possible to assess these further. Second, the cut-off for the SCL was determined based on frequency of participants having anxiety according to the STAI because there is not a specified cut-off value for the SCL-8. While this seemed like the best option, it might have led to some misclassification of anxiety in the Norwegian cohort. Also, the STAI, SCL-8 and PSS all inquire about feelings in the last two weeks to one month. While this allowed us to explore the impact of stress at different points in pregnancy, it could have introduced some misclassification as stress and anxiety could fluctuate throughout the pregnancy. Late measurements would not account for possibly higher stress or anxiety early in pregnancy, and early measurements would not consider an exposure closer to birth. While the misclassification due to the SCL cut-off is non-differential and would have left the associations found conservative, a late measurement in pregnancy might introduce an issue of reverse causality as the women could have higher levels of stress due to pregnancy complications. Third, determination of gestational age at birth is always at risk of misclassification. Since both the ultrasound scanning and report of last menstrual period happened very early in pregnancy, many weeks before the assessment of stress or anxiety, the mothers’ stress would not have influenced this misclassification. Since the misclassification is unlikely to have a specific direction, the bias would go towards the null. Fourth, even in the meta-analysis, the number of very to moderately preterm births were very low. In the perceived stress analysis, one of the cohorts, CHILD, excluded births before 35 weeks of pregnancy. Assuming that stress leads to a higher rate of very preterm births, this exclusion would most likely have resulted in an underestimation of the effects. Also, for cohorts with a later average timing of stress or anxiety measurement, some women might have given birth before the exposure assessment and thus were not included in the study.

### Interpretation

Our findings on the association between perceived stress and gestational duration are consistent with the majority of previous studies [31, 56, 58–60], but not all [32, 36]. The same applies for the association between anxiety and the length of gestation [32, 33, 61]. Additionally, the cohort-specific results in our analyses were generally aligned with each other. Both for perceived stress and anxiety, the individual cohort’s results showed the highest relative rates of giving birth in the very to moderate preterm interval (24–33 weeks), with the rates getting subsequently smaller in the later intervals. The exception to this was seen in the late preterm interval (34–36 weeks) where, both for stress and anxiety, the results were less consistent. This could be the result of chance findings. However, we found the same discrepancy in the late preterm period in an analysis of data from the Danish National Birth Cohort [62]. This may indicate that other risk factors may have more impact on the rate of giving birth during this interval than stress or anxiety.

Some previous studies have shown that the timing of stress and anxiety during pregnancy might play a role in their association with birth outcomes [33, 55, 56]. However, there is conflicting evidence regarding which trimester is the most susceptible to the potential detrimental effect of stress and anxiety. In our study, the cohorts differed in timing of measurement, varying from an average of 18 weeks of gestation in AOF to 30 weeks in MoBa. The timing of measurement in the different cohorts is important to consider in relation to inconsistencies in results, especially with regards to measurements at later gestational ages. Indeed, when measurements are performed later in the pregnancy, the associations found could possibly be the result of reverse causation as the mother could be experiencing more stress and anxiety due to already present signs of pregnancy complications leading to a possible preterm birth. While the three Canadian cohorts had measurements early enough in pregnancy to predate any early signs suggesting the risk of a possible preterm birth, EDEN, and most specifically MoBa, had measured anxiety later in pregnancy when reverse causality could be an issue. Since the estimates for the association between anxiety and length of gestation were comparable in the very to moderate preterm interval (24–33 weeks) for all three cohorts, it is likely that the association observed was genuine in this interval. The same can be concluded for the association we found between perceived stress and gestational duration in the same interval as only data from the three Canadian cohorts were used. It is however noteworthy that the only cohort in which we found an association between anxiety and the rate of giving birth in the early term interval (37–38 weeks) was MoBa. At 30 weeks of pregnancy, women could be concerned about pregnancy complications and health issues, when they had no reason to be at 18 weeks. The association found in the early term interval may thus be the result of reverse causation and warrants attention from future studies.

## Conclusion

In conclusion, by co-analyzing data from five Canadian and European cohorts, we found that both perceived stress and anxiety were associated with shorter gestation in the very to moderate preterm interval of gestational ages. The finding supports the hypothesis that stress and anxiety may account for some of the differences in preterm birth risk, although reverse causality cannot be ruled out.

### Supplementary Information

Below is the link to the electronic supplementary material.

\Supplementary file1 (DOCX 420 kb)

## Data Availability

Individual participant data used in this study are not publicly available but can be requested through the access committee of each cohort. Documentation of the harmonized dataset generated for this study is available on the Maelstrom Research website (https://maelstrom-research.org/).
