# Supplementary material for: Stress and anxiety during pregnancy and length of gestation: a federated study using data from five Canadian and European birth cohorts
Source: Eur J Epidemiol. 2024 May 28;39(7):773–83. doi: 10.1007/s10654-024-01126-4 (PMC11344005; doi:10.1007/s10654-024-01126-4)
Supplement: Supplementary file 1 — \Supplementary file1 (DOCX 420 kb) [file 10654_2024_1126_MOESM1_ESM.docx]

**Supplementary material**

Table S1: List of items used to measure stress or anxiety

| **Perceived Stress Scale** | **State Trait Anxiety Inventory** | **Symptoms Checklist-8 - Anxiety** |
| --- | --- | --- |
| In the last month, how often have you felt:  (0=Never; 1=Almost never; 2=Sometimes; 3=Fairly often; 4=Very often) | Indicate how you feel right now:  (1=Not at all; 2=Somewhat; 3=Moderately so; 4=Very much so) | Have you been bothered by any of the following during the last two weeks:  (1=Not bothered; 2=A little bothered; 3=Quite bothered; 4=Very bothered) |
| - That you were unable to control the important things in your life? - Confident about your ability to handle your personal problems? - That things were going your way? - Difficulties were pilling up so high that you could not overcome them? | - I feel calm - I feel secure - I am tense - I feel strained - I feel at ease - I feel upset - I am presently worrying over possible misfortunes - I feel satisfied - I feel frightened - I feel comfortable - I feel self-confident - I feel nervous - I am jittery - I feel indecisive - I am relaxed - I feel content - I am worried - I feel confused - I feel steady - I feel pleasant | - Feeling fearful - Nervousness or shakiness inside - Feeling tense or keyed up - Suddenly scared for no reason |

Figure S1: Selection of participants in the study

116,505

Pregnancies recruited in the 5 cohorts

5,398

Incomplete information on covariates

55,775

Complete information on covariates

5,297

Complete information on covariates

1,349

Incomplete information on covariates

6,646

Information on perceived stress (13-32 weeks)

61,173

Information on anxiety (13-32 weeks)

74,619

No information on perceived stress (13-32 weeks)

20,092

No information on anxiety (13-32 weeks)

81,265

First pregnancy in the cohorts

16,055

Second or third pregnancy in the cohorts

97,320

Singletons

19,185

Multiples

Figure S2: Directed acyclic graph for the association between stress or anxiety and gestational age at birth

Prenatal stress/anxiety

Gestational age at birth

Smoking

Education

Parity

Fertility treatment

Pregnancy complications

Infections

Congenital anomalies (child)

Age

Partner

Figure S3: Processing scripts to harmonize the maternal highest level of education according to the International Standard Classification of Education across the five cohorts

Highest level of education

0=No schooling; 2=Completed elementary; 3=Secondary not finished; 4=Completed secondary; 5=Post-secondary not finished; 6=CEGEP, college, technical, nurses training; 7=Completed university; 8=Master’s degree; 9=Doctorate; 10=Other; 88=No data; 98=Refuse to answer

*case_when(*

*a1s14q3a %in% c(0,2,3) ~3L;*

*a1s14q3a %in% c(4,5,6) ~2L;*

*a1s14q3a %in% c(7,8,9) ~1L;*

*ELSE~NA_integer_)*

What is the highest level of education you have completed?

1=Some elementary or high school (grades 1-12); 2=Graduated high school; 3=Some college/trade/university; 4=Graduated college/trade/university; 5=Some graduate school; 6=Completed graduate school

What is your highest educational level achieved?

1=Less than high school; 2=Some high school; 3=Completed high school; 4=Some college; 5=Completed college; 6=Some university; 7=Completed university, 8=Masters degree; 9=PhD; 888=Not applicable; 999=No response to this question

What is the highest level diploma you have obtained?

1=No diploma; 2=Primary school certificate; 3=Vocational training certificate (CAP); 4=Professional training certificate (BEP); 5=Vocational baccalaureate; 6=Technological baccalaureate; 7=General baccalaureate; 8=Baccalaureate diploma + 2 (DUT, BTS, DEUG); 9=Higher education diploma (2^nd^ or 3^rd^ cycle, Grande Ecole)

*recode(4:6=1 ; 2:3=2 ; 1=3 ; ELSE=NA)*

*recode(1:2=3 ; 3:4=2 ; 5=1 ; 6=2 ; 7:9=1 ; ELSE=NA)*

*if c24_niveau in (1 2) then edu_m_0 = 3;*

*else if c24_niveau in (3 4 5 6 7) then edu_m_0 = 2;*

*else if c24_niveau in (8 9) then edu_m_0 = 1;*

**Maternal highest level of education**

1 = High (ISCED 0-2), 2 = Medium (ISCED 3-4), 3 = Low (ISCED 5-8)

**3D**

**AOF**

**CHILD**

**EDEN**

Study-specific information used to generate the harmonized variable

Proposed algorithm strategy

Harmonized variable

What education do you have? (Fill in the highest level of education you have completed and current studies if you are still in school).

0=More than 1 check box filled in; 1=9-year secondary school; 2=1-2 year high school; 3=Vocational high school; 4=3-year high school general studies, junior college; 5=Regional technical college, 4-year university degree (Bachelor’s degree, nurse, teacher, engineer); 6=University, technical college, more than 4 years (Master’s degree, medical doctor, PhD)

*RECODE AA1124 (0=SYSMIS)(1=3) (2=2) (3=2) (4=2) (5=1) (6=1).*

*COMPUTE edu_m_0=AA1124.*

**MoBa**

Figure S4: Forest plot of the association between perceived stress and the rate of giving birth in intervals of gestational age without cohabitation status as a confounder*


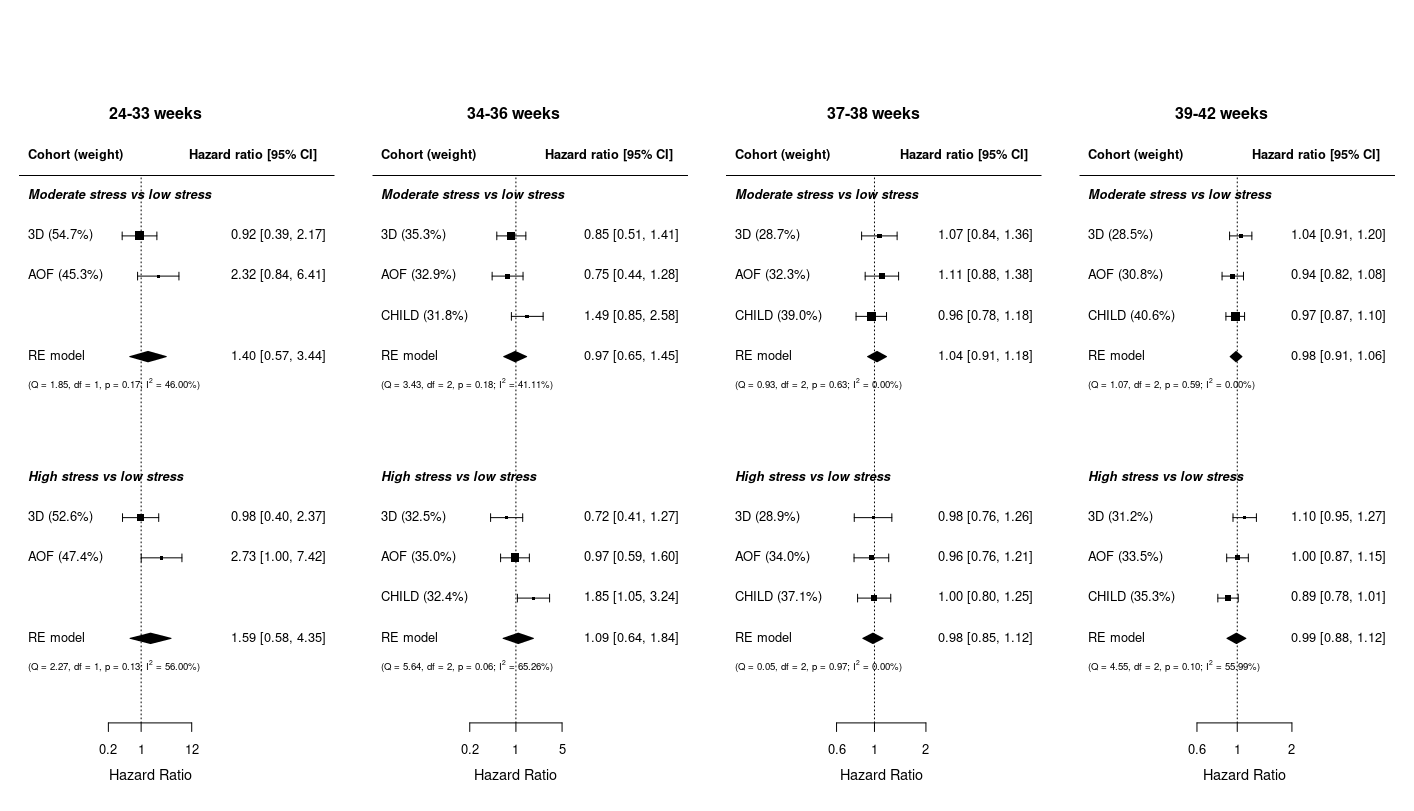


RE: Random effect

*Adjusted for maternal age, education level, parity and pregnancy complications

Figure S5: Forest plot of the association between anxiety and the rate of giving birth in intervals of gestational age without cohabitation status as a confounder*


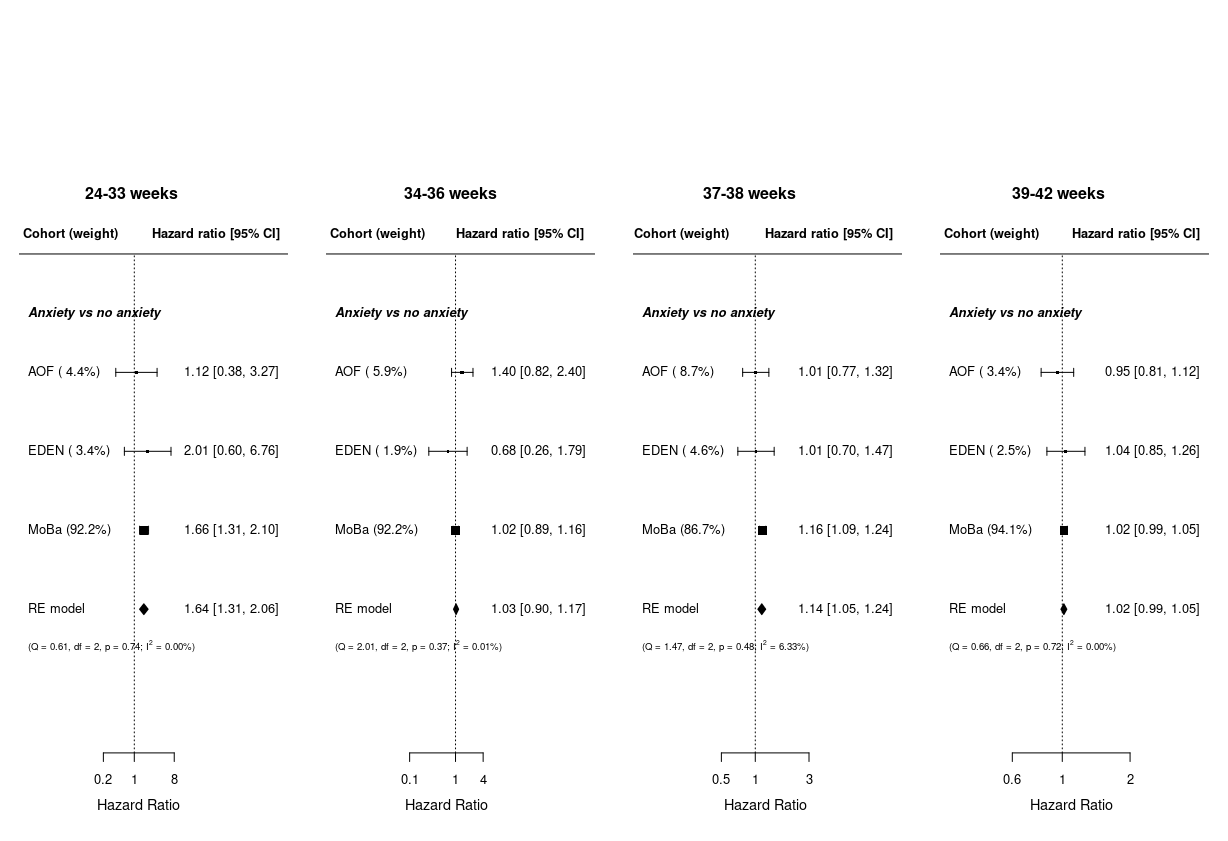


RE: Random effect

*Adjusted for maternal age, education level, parity and pregnancy complications

Figure S6: Forest plot of the association between perceived stress and the rate of giving birth in intervals of gestational age without diseases during pregnancy as a confounder*


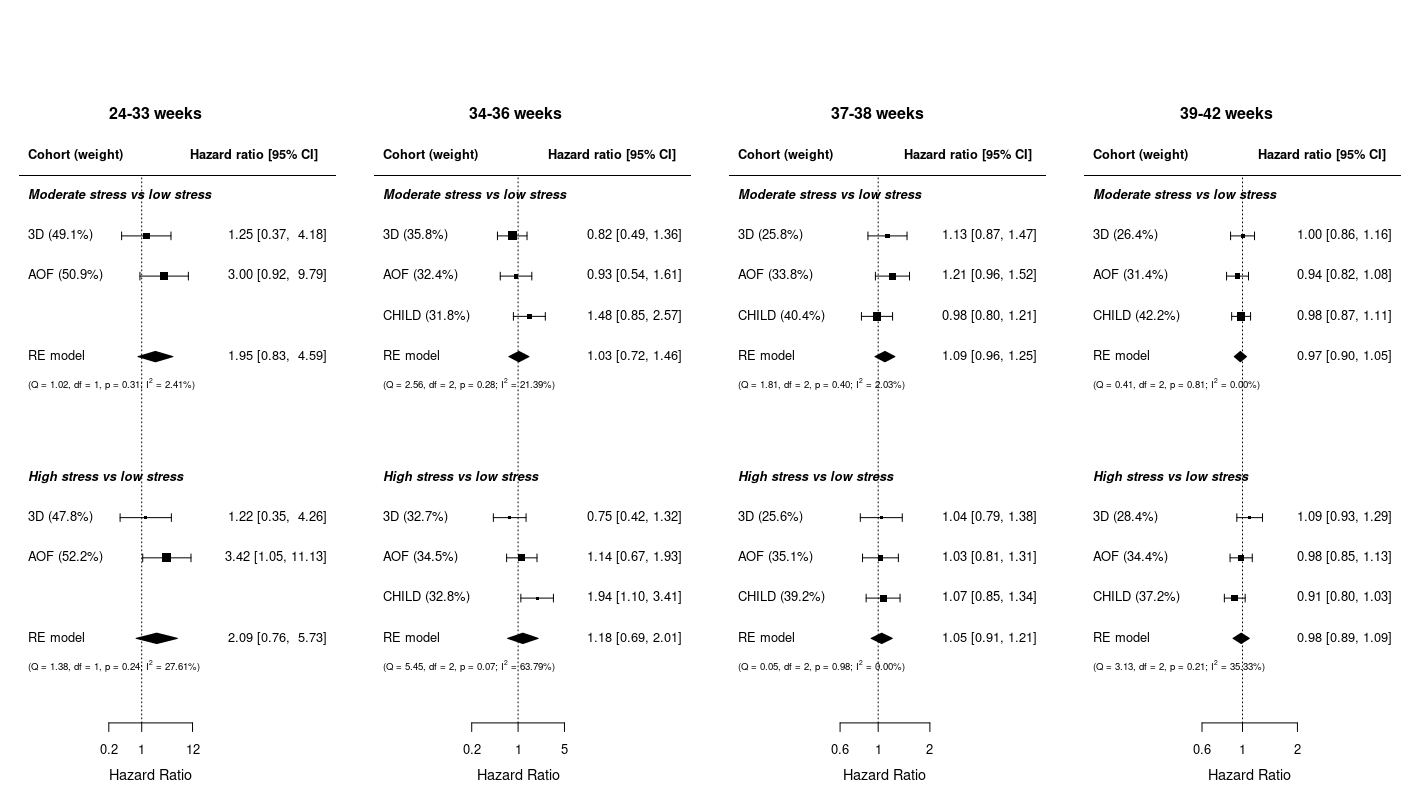


RE: Random effect

*Adjusted for maternal age, education level, cohabitation status and parity

Figure S7: Forest plot of the association between anxiety and the rate of giving birth in intervals of gestational age without diseases during pregnancy as a confounder*


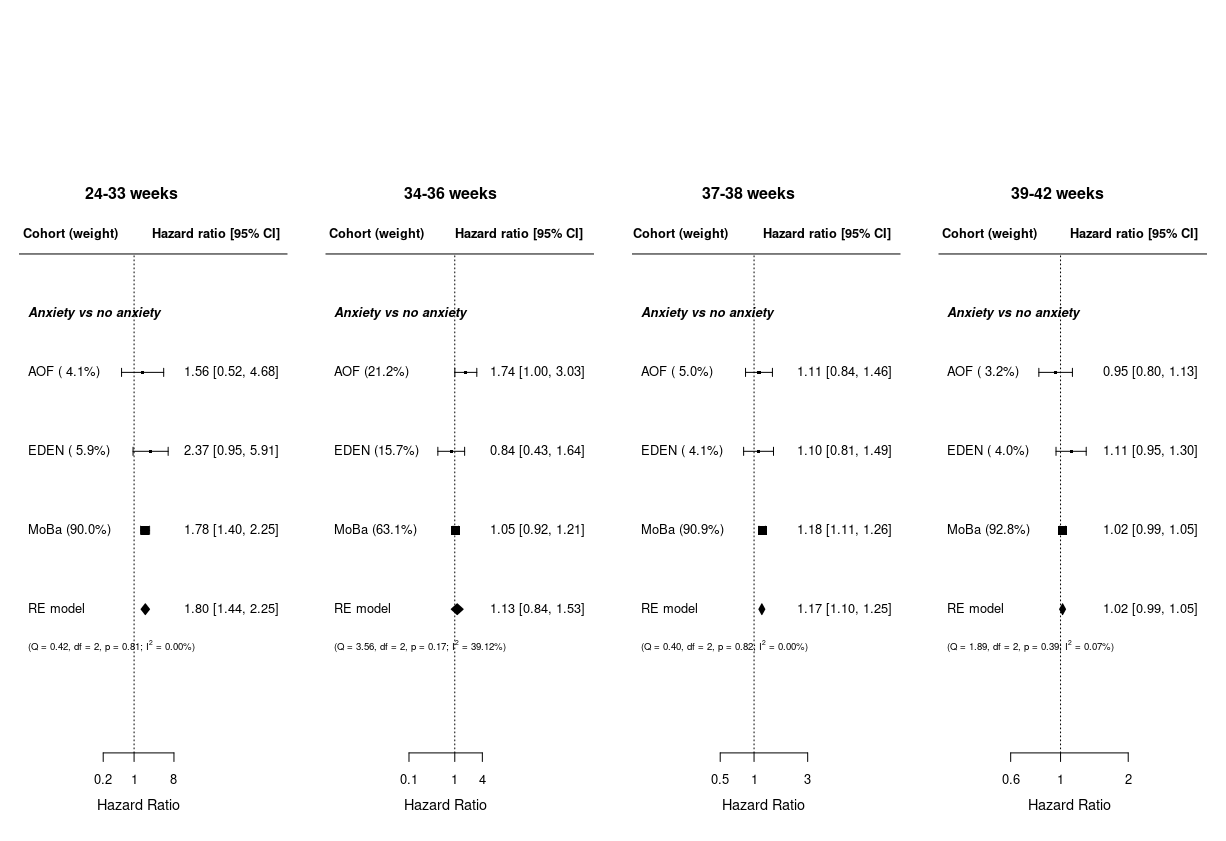


RE: Random effect

*Adjusted for maternal age, education level, cohabitation status and parity

Figure S8: Forest plot of the association between perceived stress and the rate of giving birth in intervals of gestational age without cohabitation status as a confounder in the restricted sample of participants with information on all covariates*


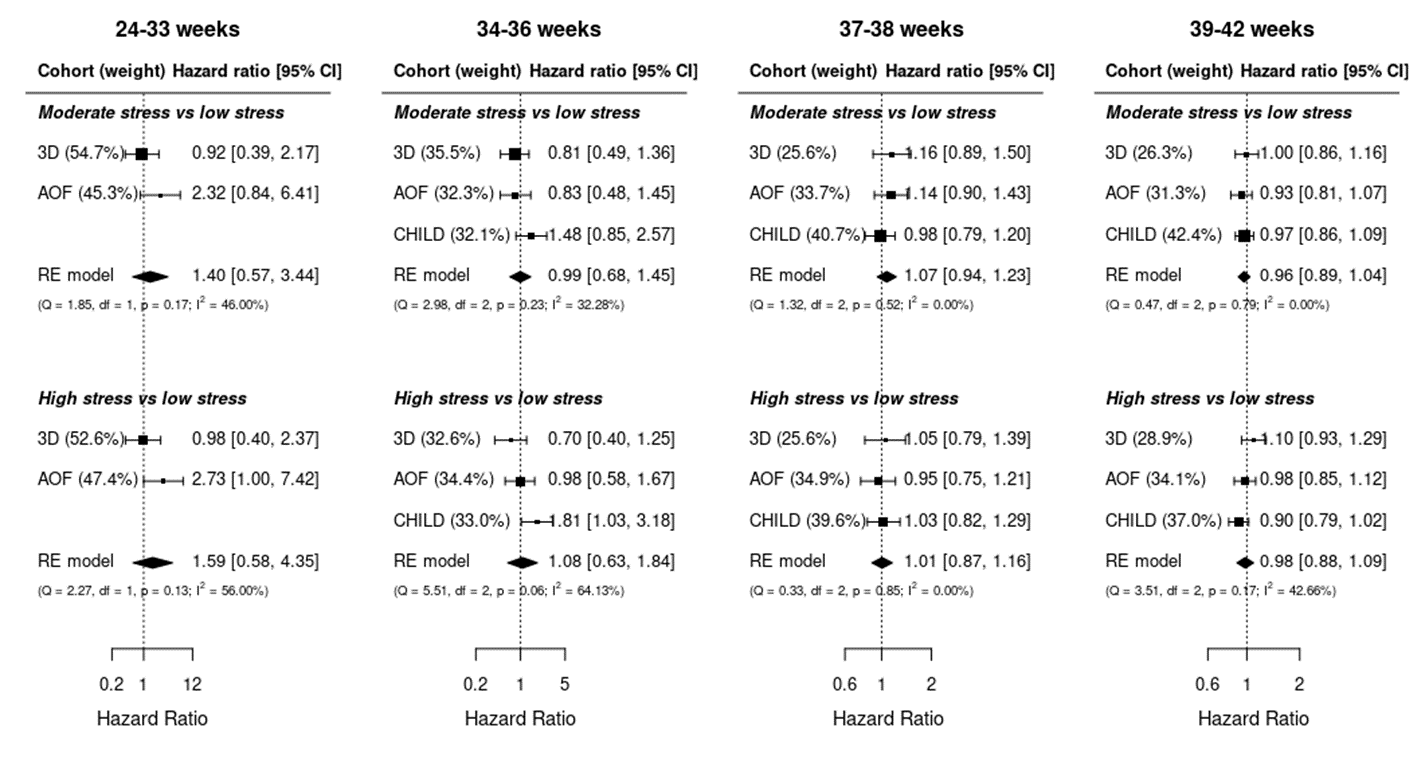


RE: Random effect

*Adjusted for maternal age, education level, cohabitation status and parity

Figure S9: Forest plot of the association between perceived anxiety and the rate of giving birth in intervals of gestational age without cohabitation status as a confounder in the restricted sample of participants with information on all covariates*


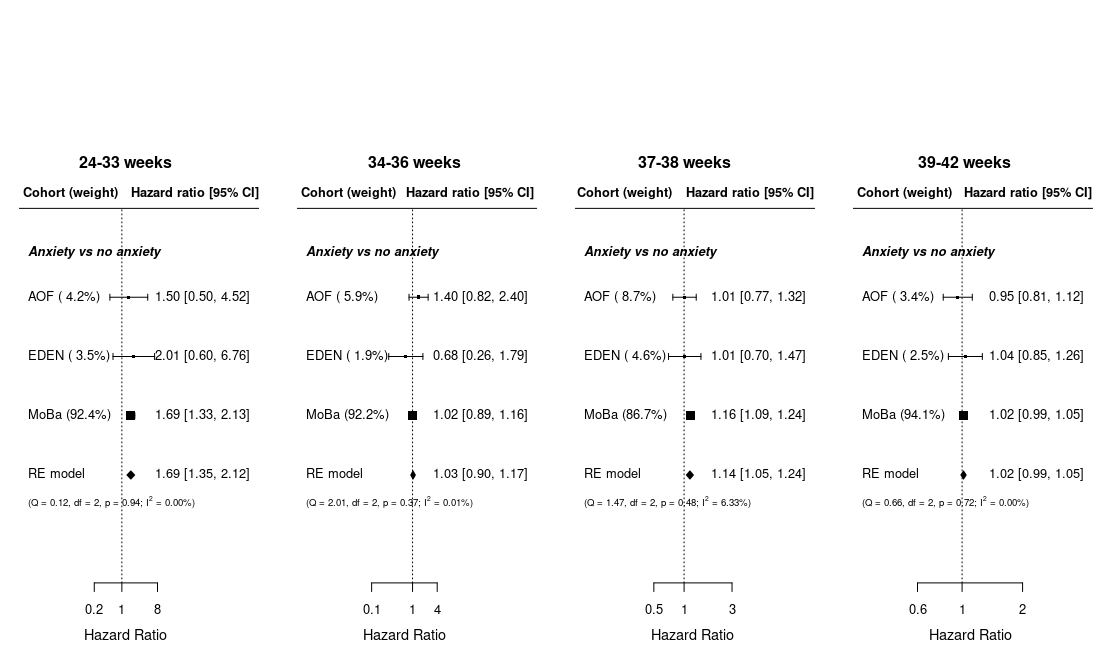


RE: Random effect

*Adjusted for maternal age, education level, cohabitation status and parity

Figure S10: Forest plot of the association between perceived stress and the rate of giving birth in intervals of gestational age without diseases during pregnancy as a confounder in the restricted sample of participants with information on all covariates*


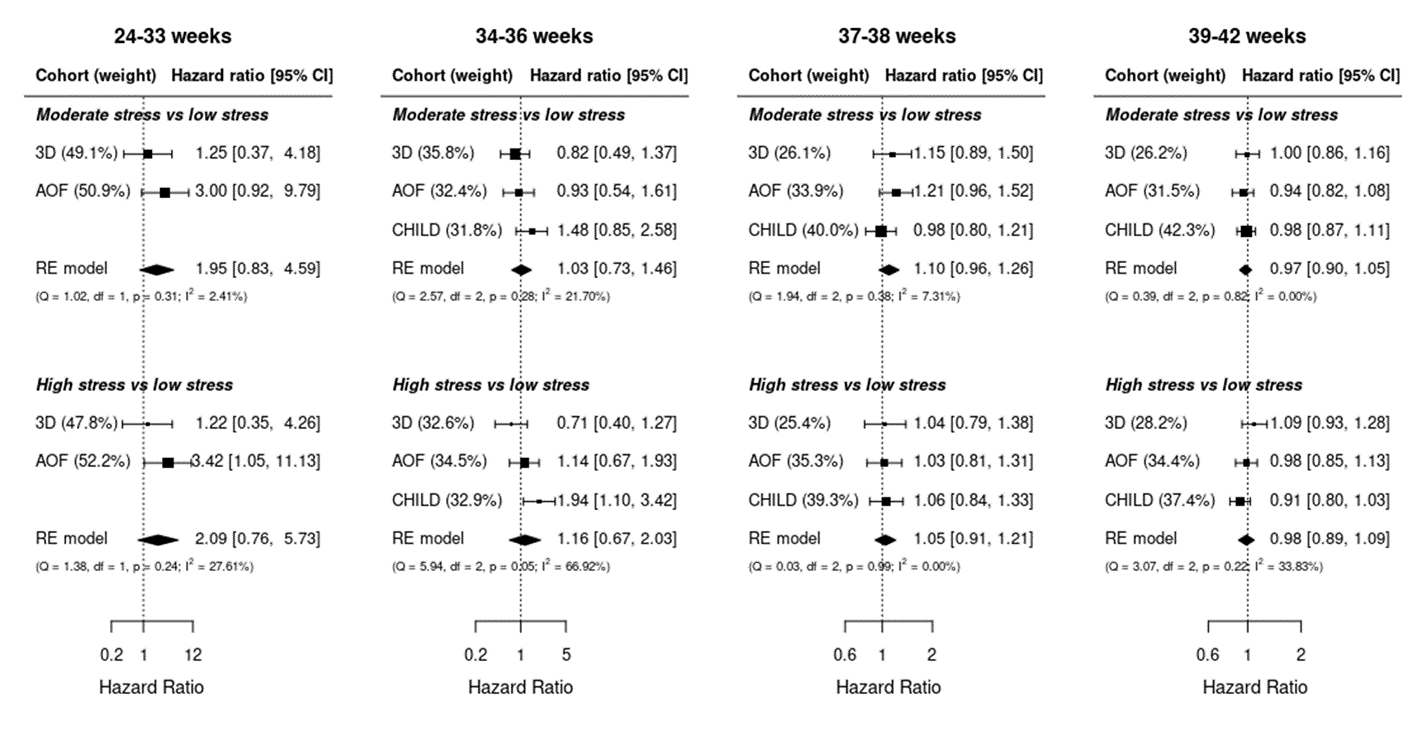


RE: Random effect

*Adjusted for maternal age, education level, cohabitation status and parity

Figure S11: Forest plot of the association between anxiety and the rate of giving birth in intervals of gestational age without diseases during pregnancy as a confounder in the restricted sample of participants with information on all covariates*


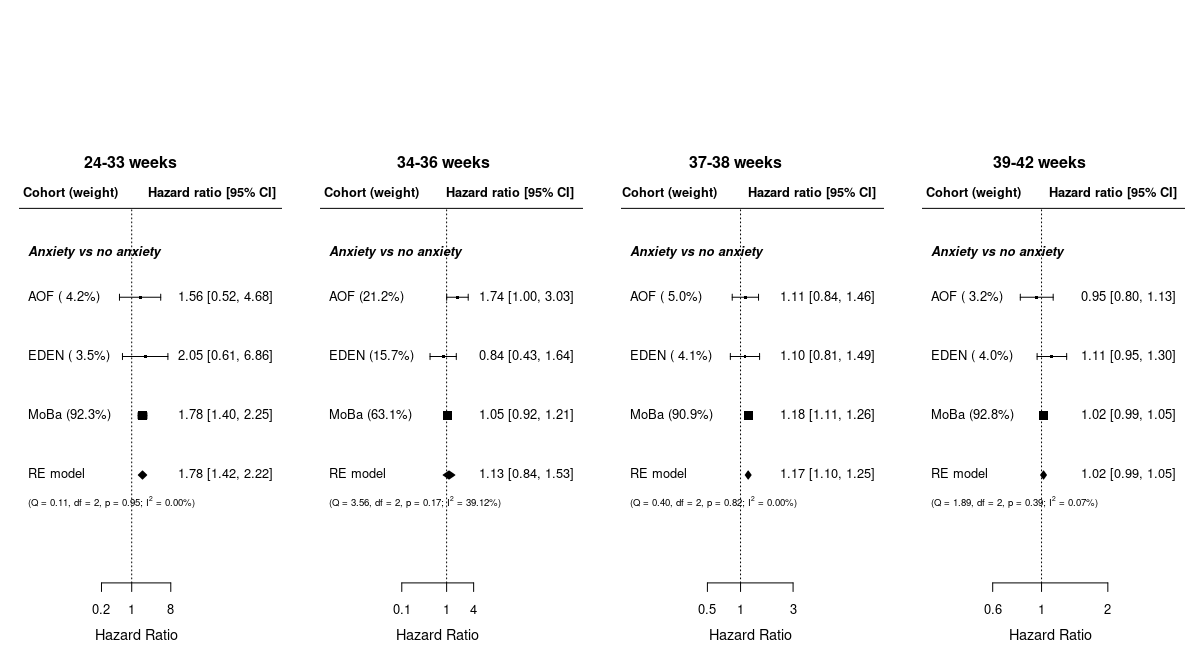


RE: Random effect

*Adjusted for maternal age, education level, cohabitation status and parity
